# The Struggling Odyssey of Infantile Primary Hyperoxaluria

**DOI:** 10.3389/fped.2021.615183

**Published:** 2021-04-20

**Authors:** Adrien Guillaume, Benedetta Chiodini, Brigitte Adams, Karin Dahan, Georges Deschênes, Khalid Ismaili

**Affiliations:** ^1^Department of Neonatology, Hôpital Erasme, Université Libre de Bruxelles (ULB), Brussels, Belgium; ^2^Department of Pediatric Nephrology, Hôpital Universitaire des Enfants Reine Fabiola, Université Libre de Bruxelles (ULB), Brussels, Belgium; ^3^Department of Genetics, Institute Pathology and Genetic (IPG), Gosselies, Belgium; ^4^Department of Pediatric Nephrology, Paris University Hospital Robert Debré, Paris, France

**Keywords:** kidney transplanation, dialysis (ESKD), hyperoxaluria type 1, infant, liver transplatation

## Abstract

**Introduction:** Oxalate overproduction in Primary Hyperoxaluria type I (PH1) leads to progressive renal failure and systemic oxalate deposition. In severe infantile forms of PH1 (IPH1), end-stage renal disease (ESRD) occurs in the first years of life. Usually, the management of these infantile forms is challenging and consists in an intensive dialysis regimen followed by a liver-kidney transplantation (combined or sequential).

**Methods:** Medical records of all infants with IPH1 reaching ESRD within the first year of life, diagnosed and followed between 2005 and 2018 in two pediatric nephrology departments in Brussels and Paris, have been reviewed.

**Results:** Seven patients were included. They reached ESRD at a median age of 3.5 (2–7) months. Dialysis was started at a median age of 4 (2–10 months). Peritoneal dialysis (PD) was the initial treatment for 6 patients and hemodialysis (HD) for one patient. Liver transplantation (LT) was performed in all patients and kidney transplantation (KT) in six of them. A sequential strategy has been chosen in 5 patients, a combined in one. The kidney transplanted as part of the combined strategy was lost. Median age at LT and KT was 25 (10–41) months and 32.5 (26–75) months, respectively. No death occurred in the series. At the end of a median follow-up of 3 years, mean eGFR was 64 ± 29 ml/min/1.73 m^2^. All patients presented retinal and bone lesions and five patients presented bones fractures.

**Conclusion:** Despite encouraging survival figures, the morbidity in IPH1 patients remains extremely heavy and its management presents a huge challenge. Thanks to the newly developed RNA-interference drug, the future holds brighter prospects.

## Introduction

Primary hyperoxaluria type I (PH1) is a rare autosomal recessive disease caused by an enzymatic defect of the alanine-glyoxylate amino-transferase, an hepatocyte peroxisomal enzyme coded by the *AGXT* gene (1, 2). In Europe, PH1 incidence is estimated at 1 per 120,000 living births (3). The infantile form of PH1 (IPH1) accounts for about 10% of PH1 cases in Europe and North America (4) and usually constitutes a life-threatening disease. It is symptomatic before the age of 1 year and quickly progresses to end-stage renal disease (ESRD) within the first 3 years of life in 80% of the patients (3, 5). In these infants, the renal clearance is exceeded by the liver production resulting in systemic oxalate accumulation (6). Oxalate depositions concern multiple organs such as the heart (7), retina (8), nervous system (9), skin (10), and bones (11, 12). The bone overload of oxalate can lead to spontaneous bone fractures but also to joint (13) and medullary (14) damages.

IPH1 has a non-specific clinical presentation, which frequently leads to a delay in diagnosis. Once suspected, the oxalate concentration in urine and blood should be measured and the disease should be confirmed by sequencing the *AGXT* gene.

Current management recommendations for IPH1 are mostly based on the 2012 guidelines of the European Hyperoxaluria Consortium (OxalEurope) (15). A conservative treatment should be started as soon as possible to slow the progression of the disease (15). Unfortunately, in IPH1 the kidney failure is often very severe at the time of diagnosis and an aggressive dialysis regimen is needed to provide maximal oxalate clearance. Nowadays the only curative option to stop the endogenous production of oxalate and restore the renal function is a double liver (LT) and kidney transplantation (KT). Depending on local practice, the two interventions can either be combined (simultaneous liver-kidney transplantation, CLKT) or sequential (liver first followed by a subsequent kidney transplantation, SLKT). The sequential procedure is usually preferred in infants for anatomical reasons and to prevent massive oxalate release from the systemic stock damaging the kidney graft (16).

New therapeutic options based on RNA interference are forthcoming. They seek to selectively silence the glycolate oxidase or the lactate deshydrogenase A enzymes in hepatocytes, thus reducing oxalate production.

Phase 3 clinical trials are now underway (17, 18), and preliminary results showed a normalization of urine oxalate excretion without significant adverse effect in most of the PH1 treated patients. This could be an important turning point as such new treatments will most probably transform the management and prognosis of the disease.

This retrospective study aims to highlight the numerous complications occurring in IPH1 affected patients and their outcome after transplantation. It illustrates the challenges and difficulties we are still facing in the management of infants affected by this condition.

## Methods

### Ethics

This study protocol, involving human patient data, was reviewed and approved by the Ethics Committee of the Queen Fabiola Children's University Hospital in Brussels (N° CEH 43/19).

We reviewed the clinical data of all infants affected by IPH1 followed at Hôpital Universitaire des Enfants - Reine Fabiola (HUDERF) in Brussels and at Hôpital Robert Debré in Paris between January 2005 and December 2018. All patients had genetically proven PH1, reached ESRD during the first year of life and were enrolled in a liver-kidney transplantation program.

The data recorded included: patient and family history, clinical, laboratory, and genetic findings as well as additional examinations such as, renal ultrasound (US), standard bone radiographies, and ophthalmic examinations. Frequency, mode and duration of dialysis (pre and post liver transplantation) have also been recorded, as well as interval between kidney and liver transplantation. Follow-up data included patient and grafts outcomes, including post-transplantation surgical and oxalosis related complications. Glomerular filtration rates have been estimated according to the Schwartz formula (19).

Oxalate depositions were assessed in the retina by an eye fundus examination. Bone involvement was defined by the presence of one or several of the following features on standard radiographies: cystic bone changes, deformations, fractures, dense metaphyseal bands, cortical thickening in long bones, and dense rims around the ossification nucleus in the calcaneum and carpal/tarsal bones (13).

Numerical results have been presented as proportions for categorical variables, means and confidence interval for normally distributed continuous variables or medians with range in case of skewed distribution.

Plasma oxalate levels (POx) of all patients were measured before and after LT. Normal distribution of the two samples was confirmed using Shapiro-Wilk test. The means were compared using Welch-Shattertwhaite *T*-test for two independent samples with unequal variance. A *p*-value <0.05 was considered statistically significant. Statistical analysis was performed using XLSTAT.

## Results

### Patient Characteristics

Seven children were included in the study, four boys and three girls. Four patients had consanguineous parents. None had known antenatal anomalies. Patients characteristics are reported in Table 1.

**Table 1:** Characteristics of included patients.

**Patient**	**Sex**	**Parental consanguinity**	**Age at RF diagnosis (months)**	**eGFR at RF diagnosis (ml/min/1,73 m^**2**^)**	**Vitamin B6 supplementation**	***AGXT* Nomenclature (NM_000030.3)/protein**
1	M	+	4.5	<15	Yes	*c.[33dupC]; [33dupC]* *p.[Pro11fs]; [Pro11fs]*
2	F	–	2	<15	Yes	*c.[358+1G>T]; [454T>A]* *p.[?]; [Phe152Ile]*
3	F	–	3	<15	Yes	*c.[642_645del]; [853G>T]* p.[Pro215fs]; [Glu285^*^]
4	M	+	3	<15	Yes	*c.[731T>C]; [731T>C]* *p.[Ile244Thr]; [Ile244Thr]*
5	M	–	5.5	<15	Yes	*c.[731T>C]; [731T>C]* *p.[Ile244Thr]; [Ile244Thr]*
6	F	+	4	<15	Yes	*c.[731T>C]; [731T>C]* *p.[Ile244Thr]; [Ile244Thr]*
7	M	+	7	30	Yes	*c.[349_350insG]; [349_350insG]* *p.[Glu117fs]; [Glu117fs]*

*RF, renal failure; eGFR, estimated glomerular filtation rate; AGXT alanine-glycoxylate aminotransferase gene*.

* *STOP CODON*.

**Figure 1:**
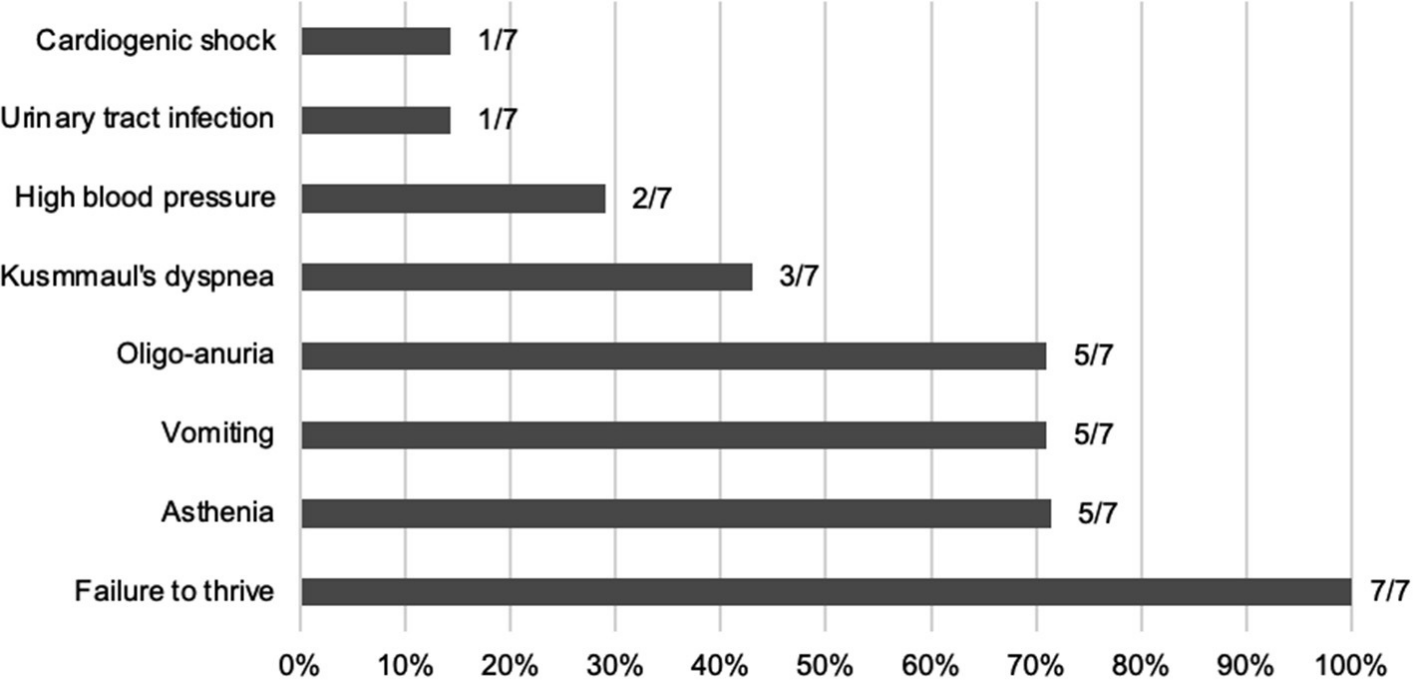
Frequency of the clinical features at diagnosis.

At time of first presentation, five children had clinical features of ESRD (oligo-anuria, vomiting, and asthenia) and all of them presented with failure to thrive (see Figure 1). They all showed poorly differentiated and hyperechogenic kidneys at first ultrasound examination compatible with nephrocalcinosis.

In all patients, PH1 was suspected at first presentation and rapidly confirmed by *AGXT* sequencing (see Table 1) except patient 1, for whom PH1 was diagnosed at the age of 14 months. At the end of the study, all patients were alive with a median age of 6.4 (3.4–12.3) years.

### Dialysis

All children started dialysis at a median age of 4 (2–10 months). Daily nocturnal peritoneal dialysis (PD) was the initial modality for six patients and hemodialysis (HD) for one. HD followed PD in four patients and was combined with PD in two children. In all patients, PD was ended after liver transplantation (LT) (see Table 2).

**Table 2:** Renal replacement therapy.

**Patient**	**Peritoneal dialysis**			**Hemodialysis**				
	**Starting age (months)**	**Duration (months)**	**Regimen**	**Starting age (months)**	**Duration (months)**	**Initial vascular access**	**Regimen**	
							**Before LT**	**After LT**
1	4.6	7.1	7 d/7	11.7	19.3	R IJV	6 ×3 h/w	5 ×3 h/w
2	2	8	7 d/7	5	21	R IJV	6 ×3 h/w	4 ×3 h/w
3	3	8	7 d/7	4.5	26.3	R IJV	6 ×3 h/w	4 ×3 h/w
4	3	2	7 d/7	5	85	R IJV	6 ×3 h/w	4 ×4 h/w
5	5.7	8.2	7 d/7	13.9	62.1	R IJV	6 ×3 h/w	4 ×4 h/w
6	4	3.9	7 d/7	7.9	67.1	R IJV	6 ×4 h/w	4 ×4 h/w
7	/	/	/	10.2	44.3	R IJV	6 ×4 h/w	3 ×4 h/w

*LT, liver transplantation; h, hour; d, day; w, week; R IJV, right intern jugular vein*.

Up to LT, HD was performed six times a week for 3–4 h. The number of HD sessions after LT was adapted to plasma oxalate levels and progressively reduced in all patients.

In six patients, dialysis was stopped after successful kidney transplantation (KT). At the end of follow-up, one patient was still on a waiting list for a kidney transplant.

### Transplantation

#### Liver Transplantation

All patients received a splitted liver graft from cadaveric donor, and one of them needed a second LT. Six transplantations were sequential and one was combined. The median age at first LT was 25 (10–41) months. The survival graft rate was of 88% after a mean follow-up of 55 ± 40 months (see Table 3).

**Table 3:** Liver and Kidney transplantations.

**Patient**	**1**	**2**	**3**	**4**	**5**	**6**	**7**
Age at LT (months)	22	10	First 11 Second 30	34	41	25	35
Weight at LT (kg)	12	8.5	First 9.6 Second 11.7	15.5	14.5	16	12.5
LT follow up (months)	19	39	First 19 Second 14	113	51	93	93
Age at KT (months)	31	26	30	First 34 Second 90	-	75	55
KT follow up (months)	9	23	14	First 2 Second 57	-	59	73
Last eGFR (ml/min/1,73 m^2^)	22	64	39	74		101	82

*LT, Liver transplantation; KT, kidney transplantation; eGFR, estimated glomerular filtration rate*.

Post-LT complications were as followed: five acute rejections, four intra-abdominal infections, one biliary leak, one biliary stenosis, and one vascular stenosis. A surgical revision was needed in five patients. In patient 3, liver graft loss occurred 19 months post-transplantation secondary to biliary and infectious complications. He received a second liver graft at the time of KT at the age of 30 months.

#### Kidney Transplantation

Six patients received a kidney graft, one of them needed a second KT (patient 4) and one is still on the waiting list (patient 5).

Median age at first KT was 33 (26–75) months. The mean interval between LT and KT was 26 ± 17 months.

At the last visit, all the transplanted children had a functioning graft after a mean follow-up of 36 ± 29 months with a mean eGFR of 64 ± 29 ml/min/1.73 m^2^.

The only kidney transplantation loss occurred in patient 4 immediately after CLKT. Biopsies showed extended tubular necrosis and ischemic glomerular lesions. He eventually received a second kidney graft at the age of 90 months.

Patient 5 developed HLA hyperimmunization after LT and is still waiting for a compatible donor.

No graft nephrocalcinosis was observed on US at the end of the follow-up.

### Plasma Oxalate Levels

**Figure 2:**
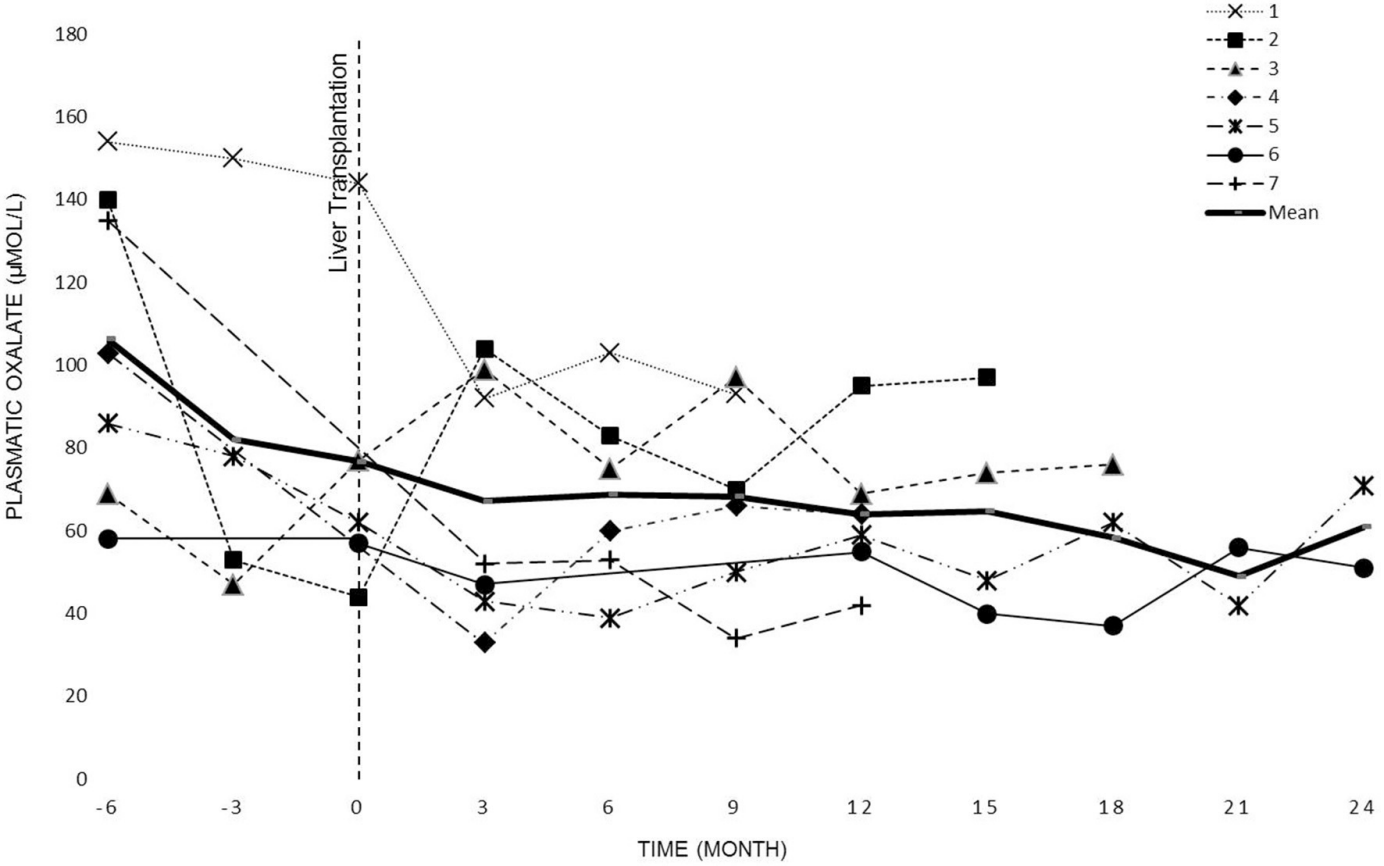
Patient's plasmatic oxalate evolution before KT.

POx levels were regularly measured in all patients before the dialysis session (see Figure 2). The overall POx mean levels before and after LT periods were of 120 ± 44 μmol/L and 79 ± 26 μmol/L, respectively. The difference was statistically significant (*p* <0.0001).

The evolution of oxalate plasmatic levels before LT up to KT or 2 years after LT (data collection stops at KT) is shown in Figure 2.

### Complications of Oxalate Depositions

From time of IPH1 diagnosis onwards, the presence of retinal and bone oxalate depositions was regularly assessed (see Table 4).

**Table 4:** Extra-renal complications of oxalosis.

**Patient**	**At diagnosis**			**During follow up**		
	**Retinal deposition**	**Bones deposition**	**Other**	**Retinal deposition**	**Bones deposition**	**Fractures**
1	+	+	–	+	+	2
2	–	–	–	+	+	9
3	–	–	–	+	+	–
4	–	–	–	+	+	–
5	–	+	–	+	+	9
6	–	+	–	+	+	1
7	–	–	–	+	+	–

Retinal oxalate depositions were observed in all patients before LT, they remained stable after LT and did not regress afterwards. Three patients developed a significant visual impairment (corrected visual acuity <5/10).

Radiographic signs of oxalate bone deposits were found in all patients. Four children presented bones fractures and two patients were particularly affected by several fractures before LT and in the first months after LT.

## Discussion

IPH1 remains a rare and severe condition. In an historical European cohort of 38 IPH1 patients, Harambat et al. (20) observed a mortality rate of 26% 3 years after initiation of dialysis.

In our series no death was observed at the end of the follow-up. However, the intensive dialysis regimen, the complications of the double organ transplantation, and the systemic oxalate deposition led to a severe morbidity.

Because antenatal anomalies were not reported in this series, no specific management was undertaken at birth, and most of the patients presented severe chronic renal insufficiency, failure to thrive and nephrocalcinosis at the time of diagnosis.

Among all seven patients, seven different *AGXT* mutations were identified (Table 1). All except one have already been described in the literature as responsible for IPH1 (class 5) (21, 22). Patient 3 is compound heterozygote for two causative variants, previously reported without precise phenotypic description (22). Only Patient 7 is homozygous for a novel change, classifying the variant as likely pathogenic (class 4). This change is an insertion variant corresponding to the insertion of a G nucleotide between nucleotides c.349 and c.350, which is a frame-shift leading the introduction of an earlier termination codon *(p.Glu117fs)*.

While waiting for liver-kidney transplantation, current guidelines recommend initiating intensive dialysis as soon as possible in order to target plasma oxalate levels below 30–45 μmol/L and avoid systemic oxalosis (3, 15). As HD provides a better oxalate clearance than PD (23), HD is recommended by the European Pediatric Working Group as the first-choice (24). However, treating small infants with chronic HD is challenging as it requires a functional vascular access and adapted equipment. Moreover, episodes of hypotension, and catheter infections or occlusions can occur (25, 26). Therefore, in our series PD was the initial dialysis modality in six out of seven patients and was combined or switched to HD as soon as possible. HD was performed six times a week, as short and frequent sessions proved to be more effective than a longer and less frequent dialysis regimen (27).

Previous studies (23, 28) have shown that even intensive dialysis regimens are insufficient to adequately remove the endogenous production of oxalate. Our experience confirms these findings. Despite intense dialysis we observed high oxalate levels in all patients (Figure 2).

It is commonly accepted that early liver-kidney transplantation is the best treatment option for IPH1 patients who have reached ESRD (15,16). Currently, there is no consensus on the best transplantation strategy between SLKT and CLKT, each strategy presenting advantages and drawbacks. In small children where anatomy may preclude a CKLT, SKLT offers the possibility of an earlier liver transplantation. However, several publications reported successful CKLT in IPH1 patients weighing 10–12 kg (29–32). In our cohort, six out of seven patients underwent a SLKT, with the aim of preserving the renal graft from new oxalate deposition, and to allow early LT even in small children. Their median weight at LT was 12 kg (the youngest was 10 months old and weighed 8.5 kg at LT). We did not observe any kidney graft loss after SLKT during the follow-up period. On the contrary, the only patient who received a CLKT experienced an immediate renal graft failure and needed a subsequent kidney transplantation.

As regard as the SKLT, the optimal interval between the two transplantations is unknown. Previous studies reported intervals from 2 to 17 months (33–41). In our patients, we were aiming to achieve POx levels as low as possible (ideally POx <20 μmol/L) (15), in order to protect the kidney graft from new oxalate deposition. However, the oxalate levels remained high with an overall mean of 79 μmol/L despite intense dialysis. Therefore, the mean interval between the two transplantations was extended to 26 months.

According to our experience, the decision about the timing of KT should probably not be based exclusively on the POx level, as this level can remain high for a too long period after LT.

Our data illustrates that even the combination of HD and PD and an early liver transplantation is not sufficient to protect against systemic oxalosis and extra-renal deposits. In our cohort, all children developed severe radiological signs of bone oxalate deposits during follow up. More than half of them presented pathological fractures, even months after LT. This is explained by the fact that both oxalate deposits and oxalate release from the bone compartment weaken the skeleton (11). We advise to thoroughly search patients for fractures and to handle the IPH1 patient with care during mobilization, both before and after LT. A good renal osteodystrophy prevention is mandatory in these patients.

The retina is also affected in IPH1. While at time of diagnosis most of our patients did not show any eye involvement, at time of LT they all showed significant oxalate retinopathy. Consistently with recent publications, oxalate retinopathy did not improve with transplantation and time (42).

Up to now, the management and the burden of IPH1 disease is extremely challenging. With the arrival of RNAi therapies, which seek to inhibit the endogenous oxalate production, a revolution in the management of IPH1 can be expected.

The avoidance of liver transplantation will probably be the most important improvement due to iRNA therapy.

Whilst it can be expected that dialysis will still be necessary for IPH1 patients with ESRD at diagnosis, the intensity of the dialysis regimen will probably be significantly lighter.

Although kidney transplantation will still be needed for PH1 patients having reached ESRD, graft survival could get closer to non-PH1 patients (20).

Finally, since the extra-renal complications are usually mild at diagnosis, we do expect an important decrease in their incidence and gravity with an early treatment by iRNA therapy.

## Conclusion

Currently, the management of IPH1 remains particularly challenging. Hopefully this will change substantially in the near future.

In the meantime, and based on our experience we propose the following approach:

- Intensive HD regimen needs to be started as soon as possible, the combination of PD and HD may be needed to maximize oxalate clearance.
- The SLKT allows an earlier LT and thus an earlier correction of the metabolic defect and should be considered as first choice.
- Plasma oxalate level should not be the only factor to define the interval between LT and KT, as it can remain above saturation threshold for several months after LT.
- Efficient fracture screening and prevention is mandatory.

New therapeutics using RNA interference could be a real game changer for IPH1 children as it could stop the endogenous oxalate production, and thus, avoid the need for LT and intensive dialysis and also prevent the extra-renal complications of oxalosis.

## Data Availability Statement

The datasets analyzed during the current study are not publicly available due to confidential reasons but are available from the corresponding author on reasonable request.

## Ethics Statement

This study protocol, involving human patient data, was reviewed and approved by the Ethics Committee of the Queen Fabiola Children's University Hospital in Brussels (N° CEH 43/19).

## Author Contributions

Material preparation, data collection, and analysis were performed by AG. The first draft of the manuscript was written by AG, BC, BA, KD, and KI. All authors contributed to the study conception and design, commented on previous versions of the manuscript, and read and approved the final manuscript.

## Conflict of Interest

The authors declare that the research was conducted in the absence of any commercial or financial relationships that could be construed as a potential conflict of interest.
